# SKI Expression Suppresses Pathogenic Th17 Cell Response and Mitigates Experimental Autoimmune Encephalomyelitis

**DOI:** 10.3389/fimmu.2021.707899

**Published:** 2021-07-15

**Authors:** Ping Li, Zengli Guo, Yisong Y. Wan

**Affiliations:** ^1^ Lineberger Comprehensive Cancer Center, University of North Carolina at Chapel Hill, Chapel Hill, NC, United States; ^2^ Department of Microbiology and Immunology, University of North Carolina at Chapel Hill, Chapel Hill, NC, United States; ^3^ Department of Rheumatology and Immunology, China-Japan Union Hospital of Jilin University, Changchun, China

**Keywords:** autoimmune disease, multiple sclerosis, experimental autoimmune encephalomyelitis, pathogenic Th17, SKI, transforming growth factor

## Abstract

Pathogenic Th17 cells are critically involved in many autoimmune diseases, while non-pathogenic Th17 cells are more immune regulatory. Understanding the mechanisms of the induction and maintenance of pathogenic Th17 cells will benefit the development of therapeutic treatments of related diseases. We have shown that the transforming growth factor-β (TGFβ) induced SKI degradation and dissociation from Smad4 complex is a prerequisite for TGFβ-induced Th17 cell differentiation. However, it is unclear whether and how SKI regulates pathogenic Th17 differentiation, which does not require TGFβ cytokine. Here we showed that SKI expression was downregulated during pathogenic Th17 cell differentiation and the ectopic expression of SKI abrogated the differentiation of pathogenic Th17 cells. Functionally, using a knock-in mouse model, we found ectopic SKI expression specifically in T cells prevented myelin oligodendrocyte glycoprotein peptide (MOG_33–55_) induced experimental autoimmune encephalomyelitis (EAE), an animal model of human multiple sclerosis. We further revealed that induced SKI expression in already differentiated pathogenic Th17 cells reduced the maintenance of Th17 program and ameliorated EAE in an adoptive T cell transfer model. Therefore, our study provides valuable insights of targeting SKI to modulate pathogenic Th17 cell function and treat Th17-related diseases.

## Introduction

IL-17-producing CD4+ T helper 17 (Th17) cells are a heterogeneous population and can be classified into pathogenic and non-pathogenic Th17 cells based on their function (1, 2). While the non-pathogenic Th17 cells are involved with tissue homeostasis (3–5), the pathogenic Th17 cells contribute to many human autoimmune diseases (6), including multiple sclerosis, inflammatory bowel disease, rheumatoid arthritis, psoriasis and type 1 diabetes (7–10). The pathogenicity of Th17 cells is influenced by both genetic and environmental factors including cytokines. While the combination of IL-6, IL-1β and IL-23 instructs the differentiation program of pathogenic Th17 cells, TGFβ1 plus IL-6 can induce the differentiation of non-pathogenic Th17 cells. However, the detailed mechanism that dictates pathogenic verses non-pathogenic Th17 program is not fully understood. The understanding of the genetic and molecular mechanisms of pathogenic Th17 cell differentiation and maintenance is important for the development of therapeutic targets to treat Th17-related diseases.

TGFβ signaling plays important roles in the development, differentiation and function of T cells (11, 12). TGFβ is required to promote non-pathogenic Th17 cell differentiation (13, 14). However, the understanding of how TGFβ signaling is regulated in pathogenic Th17 cell differentiation is evolving. It was first reported that the generation of pathogenic Th17 was independent of TGFβ1 (9). A later study showed that TGFβ3, instead of TGFβ1, was induced and important for the differentiation of pathogenic Th17 cells (10), indicating TGFβ signaling may differentially regulated in pathogenic and non-pathogenic Th17 cells. Additionally, the TGFβ superfamily member Activin has also been reported to be able to induce Th17 cells together with IL-6 (15, 16) and Activin-A-induced Th17 cells are more pathogenic than those induced by TGFβ1 (17), further diversifying the involvement of TGFβ superfamily signaling in Th17 cells. Nevertheless, underlying mechanisms of how signaling from TGFβ and its superfamily members dictate pathogenic Th17 cells and non-pathogenic Th17 cells is not fully understood. More importantly, it is unclear whether there is a central regulator that can sense and integrate TGFβ superfamily signaling to differentially modulate pathogenic and non-pathogenic Th17 cells.

We have shown that TGFβ signaling induced dissociation of SKI-Smad4 complex is a prerequisite for Th17 cell differentiation (16). As a suppressor of TGFβ signaling, SKI interacts with Smad4 to restrain the expression of ROR*γ*t, the central regulator of Th17 program (18, 19). During Th17 cell differentiation, TGFβ induces the degradation of SKI and release the inhibition on ROR*γ*t expression, which induces the Th17 program. Interestingly, our previous results show that SKI can also be regulated and degraded by Activin-A, indicating SKI could be a central regulator that can sense and integrate TGFβ superfamily signaling (16). However, whether and how SKI regulates pathogenic Th17 differentiation, which does not require TGFβ cytokine, remains unknown. Importantly, the functional importance of SKI in the pathogenic Th17 cells related autoimmune diseases remains elusive.

In this study, we investigated the role of SKI in regulating the differentiation and maintenance of pathogenic Th17 cells. We found that SKI expression was downregulated during pathogenic Th17 differentiation, which can be reversed by the TGFβ receptor inhibitor SB431542. By using a knock-in mouse model (SKI-KI) that express SKI specifically in T cells, we demonstrated that SKI expression blocked pathogenic Th17 cell differentiation but had less effect on TGFβ-induced non-pathogenic Th17 cell differentiation. Functionally, SKI-KI mice were refractory to experimental autoimmune encephalomyelitis (EAE), an animal model of human multiple sclerosis (20, 21). We further showed that ectopic expression of SKI in already differentiated pathogenic Th17 cells reduced the maintenance of Th17 program and ameliorated EAE symptoms in an adoptive T cell transfer model.

## Materials And Methods

### Animals

*Rosa26* ^iSki^, *Rag1^−/−^*, MOG_35–55_-specific 2D2 TCR-transgene, *Cd4Cre*, *ERCre* and CD45.1 congenic wild-type mice were on C57BL/6 background. *Rosa26* ^iSki^ mice were bred with *Cd4Cre* and *ERCre* mice to generate *Rosa26^iSki^ Cd4Cre* (SKI-KI) and *Rosa26* ^iSki^ *ERCre* (SKI-ER) mice, respectively. All mice were housed and bred under specific pathogen-free conditions in the animal facility at the University of North Carolina at Chapel Hill. All mouse experiments were approved by the Institution Animal Care and Use Committee of the University of North Carolina at Chapel Hill.

### Lymphocyte Isolation, T Cell Culture, and T Cell Differentiation *In Vitro*

Total CD4^+^ T cells were isolated from peripheral lymph nodes and spleens of age- and sex-matched mice and purified with CD4 microbeads (103-104-454, Miltenyi Biotec). Naïve CD4^+^ T cells were purified by naïve CD4^+^ T cell isolation kit (130-104-503, Miltenyi Biotec). Purified CD4^+^ T cells were activated with plate coated with 10 µg/ml anti-CD3 (145-2C11, BioXCell) and 10 µg/ml anti-CD28 (37.51, BioXCell) antibodies and cultured in serum-free X-VIVO 20 medium (Lonza). For pathogenic Th17 cell differentiation, 20 ng/ml IL-1β (Biolegend), 40 ng/ml IL-6 (Biolegend), 50 ng/ml IL-23 (Biolegend) and 20 µg/ml anti-IFN-γ (XMG1.2, BioXcell) were added to the culture. For TGFβ1 induced non-pathogenic Th17 cell differentiation, 2 ng/ml TGFβ1 (Biolegend), 40 ng/ml IL-6 (Biolegend) and 20 µg/ml anti-IFN-γ (XMG1.2, BioXcell) were added to the culture. For Th0 condition, 40 U/ml of IL-2 was added to the culture. TGFβ receptor I (Alk5) inhibitor SB431542 (S1067, Selleckchem) was used to block TGFβ signaling.

For the *in vitro* maintenance of pathogenic Th17 cells, cells from 4 days of pathogenic Th17 cell differentiation were harvested and transferred into 24-well plated coated with 2 µg/ml anti-CD3 and 2 µg/ml anti-CD28 antibodies and cultured in serum-free X-VIVO 20 medium for another 4 days. Samples for FACS, immunoblotting and realtime PCR were harvested at indicated time points.

### Experimental Autoimmune Encephalomyelitis

For MOG_35–55_ induced EAE, age (8–12 weeks old) and gender matched mice were subcutaneously immunized (s.c.) with 50 μg of MOG_35–55_ peptide (MEVGWYRSPFSR VVHLYRNGK, AnaSpec) and 500 μg of M. tuberculosis (Difco) emulsified in IFA (Difco). In addition, the mice were administrated 200 ng of Pertussis Toxin (List Biological Laboratories) intraperitoneally (*i.p.*) on days 0 and 2, respectively. For adoptive transferred EAE, 2D2 CD4^+^ T cells were differentiated under pathogenic Th17 differentiation conditions for 4 days and re-stimulated in fresh medium in the presence of plate-bound anti-CD3 (2 µg/ml) and anti-CD28 (2 µg/ml) antibodies for 2 days. Live cells were purified as described. Approximately 1 × 10^6^ purified cells were i.v. injected into *Rag1* ^−/−^ mice. Approximately 200 ng of Pertussis toxin (List Biological) was injected intra-peritoneal (i.p.) on days 0 and 2 after T cell transfer. For induced SKI expression in adoptive T cell transfer induced EAE, tamoxifen (TMA group) or corn oil (Control group) were injected intraperitoneally at days 1 and 3 after EAE elicitation as illustrated.

The severity of EAE was monitored and graded on a clinical score of 0 to 5 by the following standard: 0 = no clinical signs, 1 = Limp tail, 2 = Para-paresis (weakness, incomplete paralysis of one or two hind limbs), 3 = Paraplegia (complete paralysis of two hind limbs), 4 = Paraplegia with forelimb weakness or paralysis, 5 = Moribund or death. The statistical significance was analyzed by two-way multiple-range ANOVA test.

After EAE elicitation, mice were sacrificed and perfused with ice-cold phosphate buffered saline containing 20 U/ml of heparin. Spinal cords were separated from spine columns after removal of all tissues. The isolated spinal cords were minced and digested with 1 mg/ml collagenase D (Sigma) for 45 min at 37°C. The digested tissues were filtered with 40 μm strainer and centrifuged, thereafter resuspended into 38% percoll (Sigma) and centrifuged at 2,000 rpm for 20 min to separate lymphocytes. Lymphocytes were then isolated and subjected to FACS analysis.

### Flow Cytometry

Fluorescence-conjugated antibodies for CD4 (RM4-5), IFN-γ (XMG), IL-17A (TC11-18H10.1), CD8 (Ly-3) and CD45.2 (clone 104) were purchased from Biolegend and CD25 (PC61.5) and FoxP3 (FJK-16s) were from eBioscience. For FACs staining, 0.5–1 × 10^6^ cells were harvested, and surface stained followed by intracellular staining after fixation and permeabilization according the manufacture’s instruction (BD Bioscience). For intracellular cytokine staining, lymphocytes were stimulated for 4 h with 50 ng/ml of PMA (phorbol 12-myristate 13-acetate) and 1 mM ionomycin in the presence of brefeldin A. The stained cells were analyzed on LSRFortessa station (BD Biosciences) or Canto (BD Biosciences). Annexin V (BD Biosience, 550474) and 7AAD (BD Biosience, 559925) staining were used to assess apoptosis per manufacturer’s protocols.

### RNA Preparation and Realtime PCR

Total RNA was prepared from T cells using TRIzol reagent (Invitrogen) per manufacturer’s instructions and was reverse-transcribed into cDNA with iScript™ cDNA Synthesis Kit (BioRad, #1708891). Quantitative PCR was performed on ABI9700 real-time PCR system with Taqman-probe sets purchased from Applied Biosystems and Integrated DNA Technologies (IDT). The relative mRNA expression of indicated genes was calculated based on the expression of *β-actin*.

### Immunoblotting

Cells were lysed in NP40 lysis buffer (1% Nonidet P-40, 50 mM Tris (pH 7.5), 150 mM NaCl, 10% glycerol) containing protease inhibitor cocktail (Roche Molecular Biochemicals) and treated with 2× Laemmli sample buffer (Bio-Rad, 1610737) at 95°C for 10 min. The cell lysates were cleared by centrifugation at 14,000 rpm at room temperature for 15 min. Protein extracts were resolved by AnyKD SDS-PAGE gel (Bio-Rad, 4569034) and transferred to a polyvinylidene fluoride (PVDF) membrane (Millipore) and analyzed by immuno-blotting with the following antibodies: β-actin HRP, Santa Cruz (sc-4778) (WB, 1:5,000); SKI (G8, Santa Cruz), WB (1:2,000). Membranes were washed in PBS-T (1× PBS with 0.1% tween-20) and incubated with the following appropriate secondary antibodies from Jackson ImmunoResearch Laboratories: donkey anti-mouse HRP (715–035–150). The secondary antibody was used at a 1:10,000 dilution in 1× PBS-T with 5% BSA. Protein bands were visualized following exposure of the membranes to ECL substrate solution (ThermoFisher) and imaged using Image Lab software. For the original raw gel images of western blot shown in figures, see **Supplementary Figure 2**.

### Statistical Analysis

Data analysis was processed and presented by Prism (GraphPad, San Diego). Two-tailed Student’s *t*-test was used to compare two groups of samples. One-way ANOVA post Holm–Sidak’s multiple comparisons test was used to compare data with multiple groups. Two-way multiple-range ANOVA test was used to analyze clinical score in EAE experiments. A *p* value of less than 0.05 was considered significant.

## Results

### The TGFβ Signaling Was Required for the Induction of Pathogenic Th17 Cells

**Figure 1:**
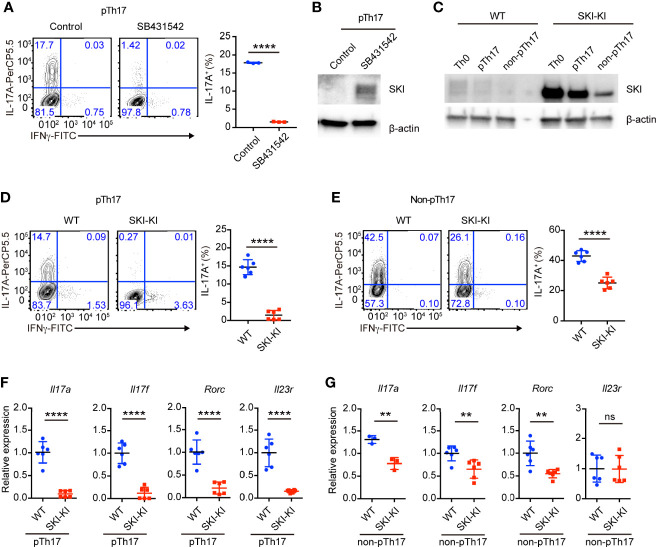
TGFβ signaling was required for pathogenic Th17 differentiation. **(A)** Flow-cytometry of IFN*γ*- or IL-17A-producing CD4^+^ cells from WT mice cultured under pathogenic Th17 (pTh17, anti-CD3/CD28 plus IL-1β, IL-6 and IL-23) polarizing conditions for four days in the presence or absence of TGFβ receptor inhibitor SB341542. Left, representative plot; right, composite data, n = 3 mice of three experiments; bars indicate the mean values, means ± s.d., ****p < 0.0001, by two-sided Student’s *t*-test. **(B)** Immunoblot analysis of SKI and β-actin in WT CD4^+^ T cells cultured under pathogenic Th17 (pTh17, anti-CD3/CD28 plus IL-1β, IL-6 and IL-23) polarizing conditions for 24 h in the presence or absence of TGFβ receptor inhibitor SB341542. Data were representative of three independent experiments. **(C)** Immunoblot analysis of SKI and β-actin in WT and SKI-KI CD4^+^ T cells cultured under Th0 (anti-CD3/CD28 plus IL-2), pathogenic Th17 (pTh17, anti-CD3/CD28 plus IL-1β, IL-6 and IL-23) and non-pathogenic Th17 (non-pTh17, anti-CD3/CD28 plus TGFβ and IL-6) polarizing conditions for 24 h. Data were representative of three independent experiments. **(D)** Flow-cytometry of IFN*γ*- or IL-17A-producing CD4^+^ cells from WT and SKI-KI mice cells cultured under pathogenic Th17 (pTh17, anti-CD3/CD28 plus IL-1β, IL-6 and IL-23) polarizing conditions for four days. Left, representative plot; right, composite data, n = 6 mice of three experiments; means ± s.d., ****p < 0.0001, by two-sided Student’s *t*-test. **(E)** Flow-cytometry of IFN*γ*- or IL-17A-producing CD4^+^ cells from WT and SKI-KI mice cells cultured under non-pTh17 (anti-CD3/CD28 plus TGFβ and IL-6) polarizing conditions for four days. Left, representative plot; right, composite data, n = 6 mice of three experiments; means ± s.d., ****p < 0.0001, by two-sided Student’s *t*-test. **(F, G)** The mRNA expression of *Il17a*, *Il17f*, *Rorc* and *Il23r* in pTh17 **(F)** and non-pTh17 **(G)** cells as in **(D)** and **(E)** respectively. Relative expression levels were measured by quantitative real-time RT-PCR and were normalized to actin expression level using the standard curve method. The results were from three independent experiments; n = 3–6 mice of three independent experiments; means ± s.d., ns, not significant, **p < 0.01, ****p < 0.0001, by two-sided Student’s *t*-test.

To understand whether TGFβ signaling is required for the differentiation of pathogenic Th17 cells, we blocked the TGFβ signaling with a pharmacological inhibitor SB431542. We found that SB431542 nearly completely suppressed the differentiation of pathogenic Th17 cells induced by cytokines IL-6, IL-1β and IL-23 **(** **Figure 1A** **)**. Our result does not contradict with the previous reports that TGFβ signaling is dispensable for the differentiation of pathogenic Th17 cells using anti-TGFβ blocking antibodies (9), as SB431542 not only targets ALK5 (TGFβ type I receptor), but also inhibits ALK4 (Activin type I receptor) and ALK7 (Nodal type I receptor, another TGFβ superfamily member) (22). Additionally, we found SB431542 treatment could drastically increase SKI protein expression **(** **Figure 1B** **)**, indicating the presence of TGFβ signaling and regulation of SKI protein level during pathogenic Th17 cell differentiation. Our result revealed that, although no TGFβ cytokine was added during pathogenic Th17 cell differentiation, the endogenous TGFβ signaling was activated from either TGFβ cytokines or its superfamily members, which could be blocked by the inhibitor SB431542.

### Ectopic Expression of SKI in SKI-KI Mice Blocked the Differentiation of Pathogenic Th17 Cells

Next, we asked whether ectopic expression of SKI in T cells is sufficient to block the generation of pathogenic Th17 cells by using *Rosa26* ^iSki^ *Cd4Cre* (SKI-KI) mice (23) that ectopically express WT SKI in T cells (**Supplementary Figure 1A**). We first examined the protein expression of SKI during pathogenic Th17 and TGFβ induced Th17 differentiation. We found while there was an increase of SKI expression in SKI-KI T cells under Th0 (IL-2 only) condition, the SKI expression was drastically downregulated in TGFβ induced non-pathogenic Th17 cells but modestly downregulated in pathogenic Th17 cells (**Figure 1C**). This result indicated that SKI functions differently in pathogenic and non-pathogenic T cells.

Indeed, the differentiation of pathogenic Th17 cells was severely blocked in SKI-KI group, and only less than 1% of IL-17A^+^ cells were generated from SKI-KI CD4^+^ T cells compared to around 15% in WT controls (**Figure 1D**). However, the blockade of Th17 cell differentiation in TGFβ-induced non-pathogenic Th17 conditions was much less efficient, with 25% of IL-17A^+^ cells in SKI-KI compared to 40% in WT controls (**Figure 1E**). The defect of Th17 differentiation in SKI-KI T cells was less likely due to cell survival, as no significant increase of apoptotic cells was observed in either pathogenic Th17 cells or non-pathogenic Th17 cells in SKI-KI group (**Supplementary Figures 1B, C**). To examine whether the severe defect of pTh17 differentiation in SKI-KI T cells is cell intrinsic or caused by inflammatory cytokines secreted by effector or memory cells, naïve CD4^+^ cells from WT (CD45.2^-^) and SKI-KI (CD45.2^+^) mice were purified, mixed together and differentiated under pathogenic Th17 conditions. Indeed, the defect of pathogenic Th17 differentiation persisted (**Supplementary Figure 1D**), indicating that SKI expression blocks pathogenic Th17 differentiation in a cell intrinsic manner. The preferential perturbation of pathogenic Th17 differentiation was further confirmed by the mRNA expression of Th17 related genes, such as *Il17a*, *Il17f*, *Rorc* and *Il23r* with real-time RT-PCR (**Figures 1F, G**). Thus, our results demonstrated that SKI expression could efficiently block the generation of pathogenic TH17 cells with less effect on TGFβ-induced non-pathogenic Th17 cells.

### SKI-KI Mice Were Refractory to EAE

**Figure 2:**
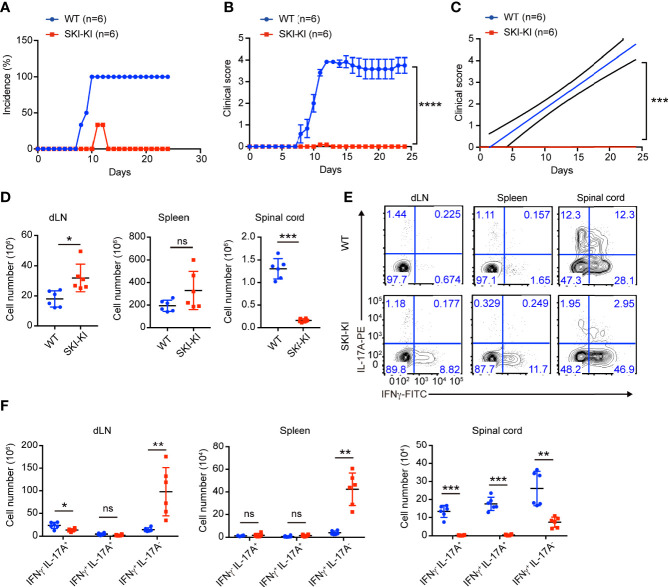
Reduced disease severity and decreased Th17 cells in SKI-KI mice during EAE. **(A–C)** The disease incidence **(A)** and the recorded clinical scores **(B)** and the linear-regression analysis **(C)** of WT and SKI-KI mice at different time points after EAE elicitation. N = 6 mice each group, representative of two independent experiments; mean ± s.e.m., ***p < 0.001, ****p < 0.0001, by two-way multiple-range ANOVA. **(D)** Total cell number of spleens, draining lymph nodes (dLN) and infiltrating cells in spinal cords of WT and SKI-KI mice at day 25 after EAE elicitation. Data were pooled of six mice per group from two independent experiments; means ± s.d.; ns, not significant, *P < 0.05, **p < 0.01, by two-sided Student’s *t*-test. **(E)** The population of IFN*γ*-, IL-17A-producing CD4^+^ T cells in draining lymph nodes (dLN, left), spleens (middle) and spinal cords (right) analyzed by flow cytometry at day 25 of EAE. The results were representative for six mice from two independent experiments. **(F)** The cell number of IFN*γ*-, IL-17A-producing CD4^+^ T cells in draining lymph nodes (dLN, left), spleens (middle) and spinal cords (right) at day 25 of EAE. Data were pooled of 6 mice from two independent experiments; mean ± s.d., ns, not significant p > 0.05, **p < 0.01, ***p < 0.001, by two-sided Student’s *t*-test.

The preferential blockade of pathogenic Th17 cells in SKI-KI T cells prompted us to investigate whether ectopic expression of SKI in T cells could inhibit EAE, where pathogenic Th17 cells are critically involved (9, 24–26). To elicit EAE, WT and SKI-KI mice were immunized with MOG_33–55_ peptide on day 0 and treated with pertussis toxin (PTX) on days 0 and 2. We found all the WT mice rapidly developed very severe EAE symptoms from 10 days after immunization, whereas SKI-KI mice showed much less incidence of EAE **(** **Figure 2A** **)**. Importantly, the average clinical score and regression clinical score were drastically lower in SKI-KI mice compared to WT controls (**Figures 2B, C**). Accordingly, much fewer infiltrating cells were recovered from spinal cords of SKI-KI with EAE compared to WT controls, although there was an increase of lymphocytes in spleens and draining lymph nodes of SKI-KI mice at day 25 of EAE **(** **Figure 2D** **)**. Further analysis of T cells in draining lymph nodes, spleens, and spinal cords of SKI-KI mice revealed that IL-17A producing T cells, but not IFNγ producing T cells, were greatly reduced in SKI-KI mice **(** **Figure 2E** **)**. Although we observed an increase of IFNγ producing T cells in the spleens and lymph nodes from SKI-KI mice, it seemed that it was not sufficient to promote disease development without Th17 cells. As a result, the number of IL-17A producing T cells, as well as IFNγ producing T cells was greatly reduced in the spinal cords of SKI-KI mice **(** **Figure 2F** **)**. Therefore, these results demonstrated that ectopic overexpression of SKI in T cells was sufficient to block pathogenic Th17-mediated autoimmunity in EAE model.

### Induced SKI Expression Was Effective to Halt Pathogenic Th17 Cell Differentiation

**Figure 3:**
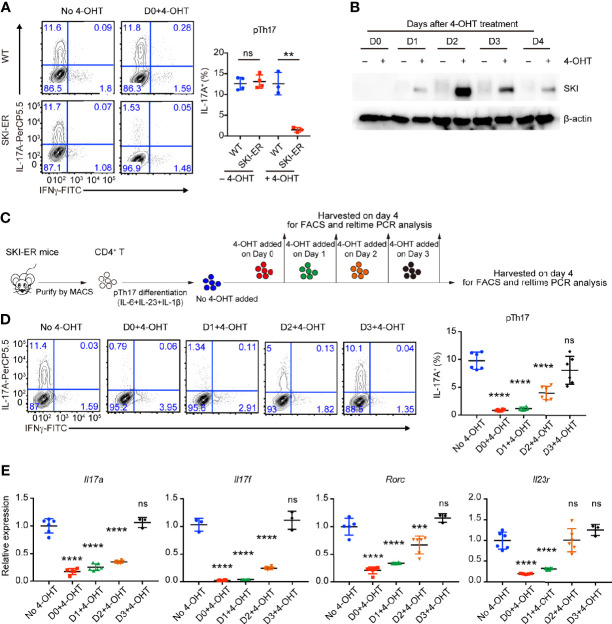
SKI expression suppressed the pathogenic Th17 cell differentiation. **(A)** Flow-cytometry of IFN*γ*- or IL-17A-producing CD4^+^ cells from WT and SKI-ER mice cultured under pTh17 (anti-CD3/CD28 plus IL-1β, IL-6 and IL-23) polarizing conditions with or without 4-hydroxy-tamoxifen for four days. Left, representative plot; right, composite data from three to four mice per group of three independent experiments; means ± s.d., ns, not significant p > 0.05, **p < 0.01, by two-sided Student’s *t*-test. **(B)** Immunoblot analysis of SKI and β-actin in SKI-ER CD4^+^ T cells cultured under pTh17 polarizing conditions added with 4-hydroxytamoxifen (4-OHT) for indicated days as shown. Data were representative of three independent experiments. **(C)** Schematic diagram of inducing SKI expression at different days during pTh17 differentiation in SKI-ER CD4^+^ T cells with 4-hydroxytamoxifen (4-OHT). **(D)** Flow-cytometry of IFN*γ*- or IL-17A-producing CD4^+^ cells from SKI-ER mice cultured under pTh17 polarizing conditions for four days, with 4-hydroxy-tamoxifen added into cell culture at indicated days. Left, representative plot; right, composite data from 6 mice per group of three independent experiments; means ± s.d., ns, not significant p > 0.05, ****p < 0.0001, by one-way ANOVA post Holm–Sidak’s multiple comparisons test. **(E)** The mRNA expression of *Il17a*, *Il17f*, *Rorc* and *Il23r* in pTh17 cells as in **(A)**. Relative expression levels were measured by quantitative real-time RT-PCR and were normalized to actin expression level using the standard curve method. The data were pooled from three to six mice per group of three independent experiments, n = 3–6 mice; means ± s.d., ns, not significant p > 0.05, ***p < 0.001, ****p < 0.0001, by one-way ANOVA post Holm–Sidak’s multiple comparisons test.

As *Cd4Cre* mediated SKI activation occurred in early T cell development in *Rosa26* ^iSki^ *Cd4Cre* (SKI-KI) mice, all naïve SKI-KI T cells already expressed high level of SKI before T cell activation and differentiation (**Supplementary Figure 1A**), making it difficult to study the inhibition role of SKI during pathogenic Th17 cell differentiation. To understand whether SKI could suppress pathogenic Th17 differentiation after T cell activation, we generated the *Rosa26* ^iSk^ *^i^ ERCre* (SKI-ER) mice by breeding *Rosa26* ^iSki^ mice with ERCre mice (27), so that the expression of SKI can be induced at the time of our choice with tamoxifen *in vitro* and *in vivo* (27). While comparable pathogenic Th17 cells were generated in SKI-ER group in the absence of 4-hydroxytamoxifen (4-OHT), 4-OHT treatment at day 0 effectively abrogated pathogenic Th17 cell differentiation in SKI-ER but not in WT controls (**Figure 3A**), consistent with the increased protein expression of SKI in SKI-ER T cells as early as one day after 4-OHT treatment (**Figure 3B**).

Next, we induced SKI expression at different time points during ongoing pathogenic Th17 differentiation by adding 4-OHT at the indicated time points (**Figure 3C** **)**. We found that SKI induction within 48 hours of T cell activation was sufficient to halt the ongoing differentiation of pathogenic Th17 cells (**Figures 3D, E**). Yet, no obvious suppression was observed when SKI was induced at 72 hours of T cell activation (**Figures 3D, E**), which could be due to insufficient time to allow effective SKI expression in SKI-ER T cells.

### SKI Expression Impaired the Maintenance of Pathogenic Th17 Cells *In Vitro*

**Figure 4:**
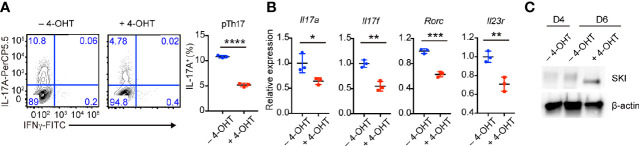
Induced SKI expression disrupted the *in vitro* maintenance of pathogenic Th17 cells. **(A)** Flow-cytometry of IFN*γ*- or IL-17A-producing pTh17 cells from SKI-ER mice treated with or without 4-hydroxytamoxifen (4-OHT). The pTh17 cells were first generated under pTh17 polarizing conditions for four days and 4-hydroxytamoxifen (4-OHT) was added for another four days. Left, representative plot; right, composite data from 3 mice per group of three independent experiments; means ± s.d., ****p < 0.0001, by two-sided Student’s *t*-test. **(B)** The mRNA expression of *Il17a*, *Il17f*, *Rorc* and *Il23r* in pTh17 cells as in **(A)**. The pTh17 cells were first generated under pTh17 polarizing conditions for four days and 4-hydroxytamoxifen (4-OHT) was added for another two days. Relative expression levels were measured by quantitative real-time RT-PCR and were normalized to actin expression level using the standard curve method. The data were from n = 3 mice per group of three independent experiments; means ± s.d., *p < 0.05, **p < 0.01, ***p < 0.001, by two-sided Student’s *t*-test. **(C)** Immunoblot analysis of SKI and β-actin in SKI-ER pTh17 cells at days 4 and 6 treated with or without 4-hydroxytamoxifen (4-OHT). The images were representative of three independent experiments.

To further investigate whether SKI controls the maintenance of already differentiated pathogenic Th17 cells, we first generated pathogenic Th17 cells from SKI-ER T cells without 4-OHT treatment. Then we induced SKI expression in these cells by adding 4-OHT. After additional four days of culture, we found there was a decrease of the percentage of IL-17^+^ T cells in 4-OHT treated group compared to the control group (**Figure 4A**). Moreover, we observed a downregulation of *Il17a*, *Il17f*, *Rorc* and *Il23r* in pathogenic Th17 cells two days after 4-OHT treatment by real-time RT-PCR **(** **Figure 4B**), coinciding with induced SKI expression (**Figure 4C**). These findings suggest that SKI suppressed not only the induction but also the maintenance of pathogenic Th17 cells.

### Induced SKI Expression Impaired the Maintenance of Pathogenic Th17 Cells and Mitigated Pathogenic Th17 Cell Elicited EAE

**Figure 5:**
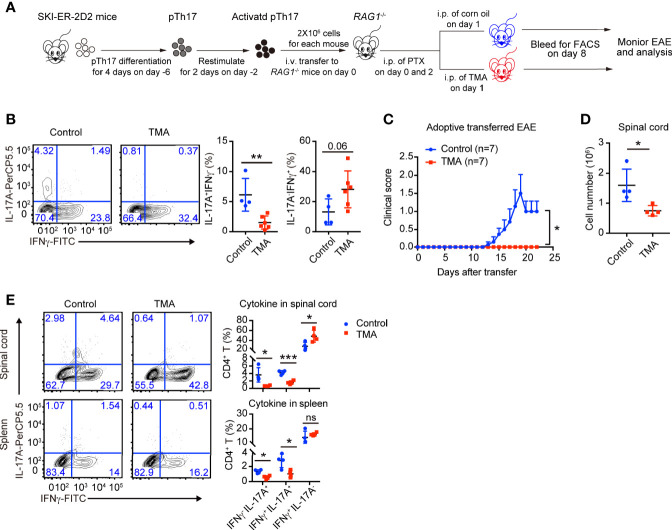
Induced SKI expression disrupted the *in vivo* maintenance of pathogenic Th17 cells and ameliorated pathogenic Th17 cells-elicited EAE. **(A)** Schematic diagram of testing the *in vivo* maintenance and function of SKI-ER-2D2 pathogenic Th17 cells using adoptive T cell transfer model. **(B)** Flow-cytometry of IFN*γ*- or IL-17A-producing CD4^+^ T cells from blood of *Rag1* ^−/−^ recipient mice transferred with SKI-ER-2D2 pTh17 cells and treated with tamoxifen (TMA) or vehicle control (Control) for 8–10 days. Left, representative plot; right, composite data, n = 4 mice for control group, n = 6 for TMA group of two independent experiments; means ± s.d., ns, not significant p > 0.05, **p < 0.01, by two-sided Student’s *t*-test. **(C)** The EAE clinical scores of the mice that received SKI-ER-2D2 pTh17 cells and treated with tamoxifen (TMA) or vehicle control (Control). The data were from two independent experiments; n = 7 mice in each group, mean ± s.e.m., *p <0.05 per two-way multiple-range ANOVA test. **(D)** Cellularity of infiltrating cells in spinal cords of EAE mice with tamoxifen (TMA) or vehicle control (Control) treatment at day 22 after EAE elicitation. Data are pooled of four mice per group from two independent experiments; means ± s.e.m.; *P < 0.05, by two-sided Student’s *t*-test. **(E)** The population of IFN*γ*-, IL-17A-producing CD4^+^ T cells in spinal cords (upper) and spleens (bottom) from mice at day 22 of adoptive transferred induced EAE. The results were representative of two independent experiments; n = 4 mice, mean ± s.d., ns, not significant p > 0.05, * p < 0.05, ***p < 0.001 by two-sided Student’s *t*-test.

The *in vitro* results encouraged us to examine whether SKI also controls the maintenance of pathogenic Th17 *in vivo* and if so whether induced SKI expression can ameliorate Th17-related diseases. We generated *Rosa26* ^iSk^ *^i^ ERCre 2D2* (SKI-ER-2D2) mice, where T cells harbor MOG_33–55_ specific TCR transgene (28). SKI-ER-2D2 pathogenic Th17 cells were differentiated and then transferred to *Rag1* ^−/−^ mice to elicit EAE (**Figure 5A** **)** (29). Tamoxifen was then administrated into recipient mice *via* intra-peritoneal injection (i.p.) to induce SKI expression in transferred SKI-ER-2D2 pathogenic Th17 cells. We found that IL-17^+^ T cells, but not IFN*γ*+ T cells, were reduced in the blood from tamoxifen treated mice compared to the controls 8 days after T cell transfer (**Figure 5B**), indicating SKI expression suppressed the maintenance of already differentiated pathogenic Th17 cells *in vivo*. Importantly, tamoxifen treatment effectively reduced the clinical scores of EAE induced by the transferred SKI-ER-2D2 pathogenic Th17 cells (**Figure 5C**) and suppressed the immune cell infiltration in spinal cords **(** **Figure 5D** **)**. Further analysis revealed that tamoxifen treatment suppressed the population of IL-17^+^IFN*γ* ^-^ and IL-17^+^IFN*γ* ^+^ T cells in both spleens and spinal cords. In contrast, IL-17^-^IFN*γ* ^+^ population was slightly increased in spinal cords **(** **Figure 5E** **)**, indicating SKI expression specifically affected Th17 cell population during EAE. In summary, our results showed that SKI induction in T cells can disrupt pathogenic Th17 cell response and to mitigate Th17 cell promoted disease.

## Discussion

Pathogenic Th17 cells drive the pathogenesis of many autoimmune diseases, including multiple sclerosis, inflammatory bowel disease, rheumatoid arthritis, psoriasis, and type 1 diabetes (7–10). The study of the regulation of pathogenic Th17 cell differentiation and maintenance will provide novel insights into the development of therapeutic targets to treat these diseases. Unlike the well-established requirement of TGFβ in non-pathogenic Th17 cells (3, 10), the role of TGFβ signaling in regulating pathogenic has been evolving. Evidence suggests that other TGFβ superfamily members such as TGFβ3 and Activin, instead of TGFβ1; may be critical for the differentiation of pathogenic Th17 cells (10, 17). It is important to address the question of how different signaling from TGFβ family members are relayed by downstream pathways to control the functionally distinct pathogenic and non-pathogenic Th17 cells. In this study, we showed that ectopic expression of SKI, a suppressor of TGFβ signaling, in T cells could efficiently block pathogenic Th17 cell differentiation and the development of EAE, but only partially affected TGFβ-induced non-pathogenic Th17 cells. We further showed that induced expression of SKI in already differentiated pathogenic Th17 cells impaired their maintenance and function in adoptive transferred EAE. Therefore, our study provided novel insights of how the TGFβ signaling could be differentially regulated by SKI to modulate the differentiation and maintenance of pathogenic Th17 cells.

Many approaches targeting TGFβ signaling have been developed, including blocking antibodies, dominant negative mutations, and small molecule inhibitors (30–32). However, its application to Th17 cells is very limited. One reason could be that the TGFβ signaling plays very important roles in many aspects of T cell biology including development, differentiation and homeostasis (11, 12, 33). Since there is a lack of clear molecular target that is specifically required for pathogenic Th17 cells, blocking TGFβ signaling also affect the other immune cells, especially Treg cells, resulting in systemic lethal autoinflammation and autoimmunity. By using the *Rosa26^iSki^ Cd4Cre* (SKI-KI) mouse strain, we were able to efficiently block the differentiation of pathogenic Th17 cells, but only partially disrupted the TGFβ-induced non-pathogenic Th17 cells. In addition, Treg cell populations are unperturbed in SKI-KI mice which are phenotypically normal without developing systemic autoimmune syndrome (23). These results revealed that SKI could be a new target to fine tune TGFβ superfamily signaling to specifically modulate pathogenic TH17 cells. Thus, our study provides important clues to manipulate TGFβ signaling for therapeutic purposes.

Our study reveals a critical role of SKI in regulating pathogenic Th17 cells and related autoimmune disease. The further study of the detailed molecular regulation of SKI protein will provide more clues of how SKI can be targeted by therapeutic approaches, in additional to ectopic expression. While the expression of SKI protein is negatively regulated by TGFβ signaling, the regulation is predominantly *via* posttranslational modification and ubiquitination mediated degradation (16, 33, 34). Our study has shown that both TGFβ and Activin could induce the degradation of SKI protein in T cells (16). However, the detailed pathway that relays the signaling from TGFβ receptor to polyubiquitination and proteasome-mediated protein degradation remains elusive. While the E3 ubiquitin ligases Arkadia is reported to target SKI in non-lymphoid cells (34, 35), whether it also regulates SKI stability in T cells remains to be examined. Additionally, whether there are other E3 ubiquitin ligases and proteasomal-independent mechanisms exist to modulate SKI expression need to be further investigated.

In summary, we demonstrated that expression of SKI could preferentially block the induction and maintenance of pathogenic Th17 cells, with less effect on the TGFβ-induced non-pathogenic Th17 cells. We showed that the mouse models that express WT SKI specifically in T cells were refractory to pathogenic Th17 cell induced EAE. This study suggests targeting SKI could be a viable approach to control pathogenic Th17 cell response and to treat Th17 cell related diseases.

## Data Availability Statement

The original contributions presented in the study are included in the article/**Supplementary Material**. Further inquiries can be directed to the corresponding authors.

## Ethics Statement

The animal study was reviewed and approved by Institution Animal Care and Use Committee of the University of North Carolina at Chapel Hill.

## Author Contributions

PL and ZG contributed equally to this manuscript PL and ZG designed and performed the experiments. ZG analyzed the data and wrote the manuscript. YYW oversaw project completion, secured funding, and contributed to the manuscript. All authors contributed to the article and approved the submitted version.

## Funding

This study was supported by the NIH (AI123193, AI160774) and the National Multiple Sclerosis Society (RG-1802-30483) for YYW.

## Conflict of Interest

The authors declare that the research was conducted in the absence of any commercial or financial relationships that could be construed as a potential conflict of interest.

## Supplementary Material

Supplementary Figure 1 SKI expression, apoptosis and pTh17 cell differentiation of T cells from SKI-KI mice **(A).** Immunoblot analysis of SKI and β-actin in naïve CD4^+^ T cells from WT and SKI-KI mice. Images were representative of three independent experiments. **(B)** Apoptosis of WT and SKI-KI pTh17 cells cultured at day was analyzed by flow-cytometry using Annexin V and 7AAD staining. Left, representative sample; right, statistical summary of four replicates; means ± s.d.; ns, not significant, by two-sided Student’s *t*-test. **(C)** Apoptosis of WT and SKI-KI cTh17 cells cultured at day was analyzed by flow-cytometry using Annexin V and 7AAD staining. Left, representative sample; right, statistical summary of four replicates; means ± s.d.; ns, not significant, by two-sided Student’s *t*-test. **(D)** Flow-cytometry of IFN*γ*- or IL-17A-producing CD4^+^ cells from mixed naïve WT (CD45.2^-^) and SKI-KI (CD45.2^+^) cells cultured under pathogenic Th17 (pTh17, anti-CD3/CD28 plus IL-1β, IL-6 and IL-23) polarizing conditions for four days. Left, representative plot; right, statistical summary of four replicates; means ± s.d., *****p* < 0.0001, by two-sided Student’s *t*-test.

Supplementary Figure 2 Uncropped gel images with size marker indications.This file contains **Supplementary Figures 2**: Uncropped western blot images.
