# Supplementary figures and images for: SKI Expression Suppresses Pathogenic Th17 Cell Response and Mitigates Experimental Autoimmune Encephalomyelitis
Source: Front Immunol. 2021 Jul 15;12:707899. doi: 10.3389/fimmu.2021.707899 (PMC8321777; doi:10.3389/fimmu.2021.707899)

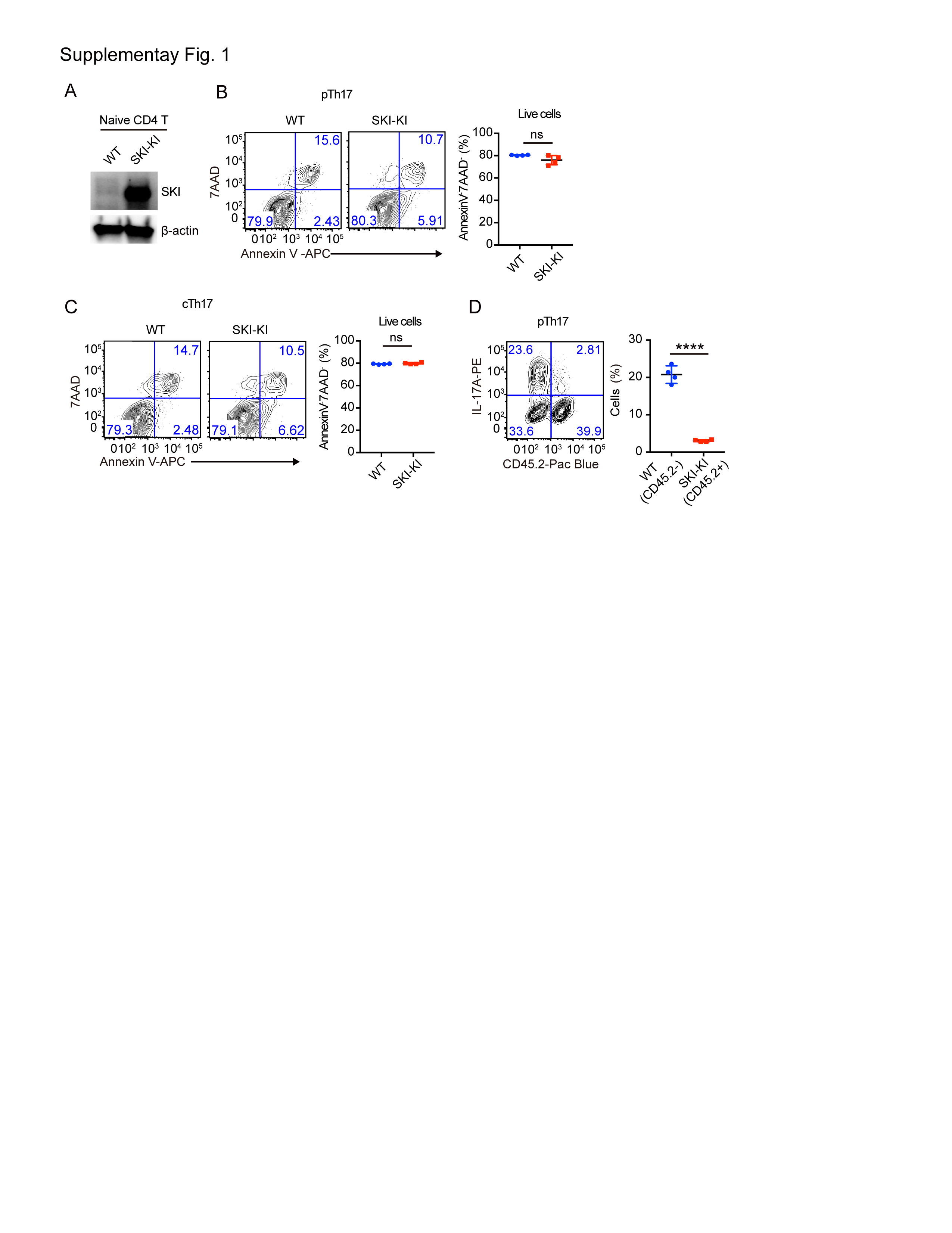

Supplement: Supplementary Figure 1 — SKI expression, apoptosis and pTh17 cell differentiation of T cells from SKI-KI mice (A). Immunoblot analysis of SKI and β-actin in naïve CD4+ T cells from WT and SKI-KI mice. Images were representative of three independent experiments. (B) Apoptosis of WT and SKI-KI pTh17 cells cultured at day was analyzed by flow-cytometry using Annexin V and 7AAD staining. Left, representative sample; right, statistical summary of four replicates; means ± s.d.; ns, not significant, by two-sided Student’s t-test. (C) Apoptosis of WT and SKI-KI cTh17 cells cultured at day was analyzed by flow-cytometry using Annexin V and 7AAD staining. Left, representative sample; right, statistical summary of four replicates; means ± s.d.; ns, not significant, by two-sided Student’s t-test. (D) Flow-cytometry of IFNγ- or IL-17A-producing CD4+ cells from mixed naïve WT (CD45.2-) and SKI-KI (CD45.2+) cells cultured under pathogenic Th17 (pTh17, anti-CD3/CD28 plus IL-1β, IL-6 and IL-23) polarizing conditions for four days. Left, representative plot; right, statistical summary of four replicates; means ± s.d., ****p < 0.0001, by two-sided Student’s t-test. [file Image_1.tif]
