# Supplementary material for: SKI Expression Suppresses Pathogenic Th17 Cell Response and Mitigates Experimental Autoimmune Encephalomyelitis
Source: Front Immunol. 2021 Jul 15;12:707899. doi: 10.3389/fimmu.2021.707899 (PMC8321777; doi:10.3389/fimmu.2021.707899)

Supplementay Fig. 2

1. Full unedited gel for Figure 1B

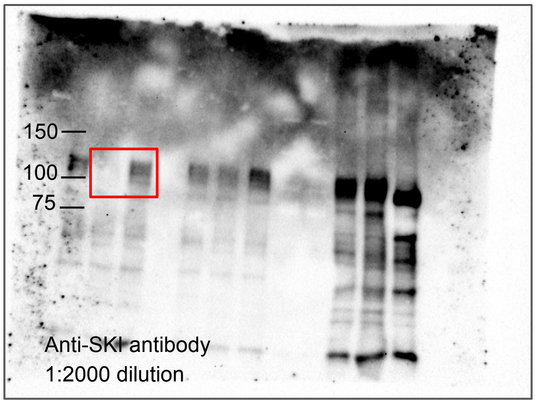

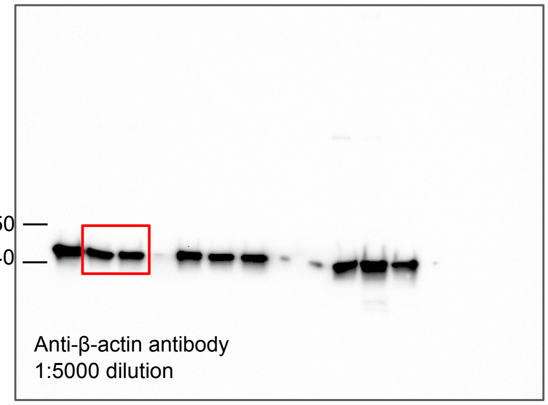

2. Full unedited gel for Figure 1C and Supplementary Figure 1A

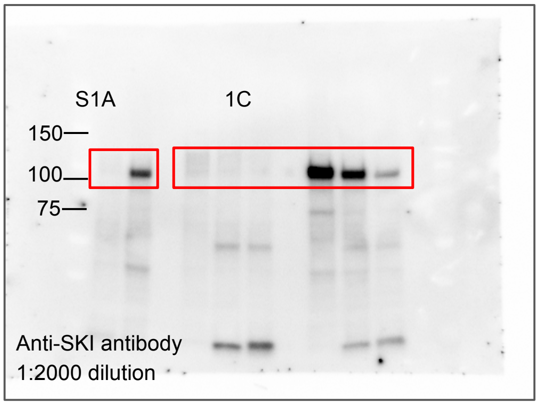

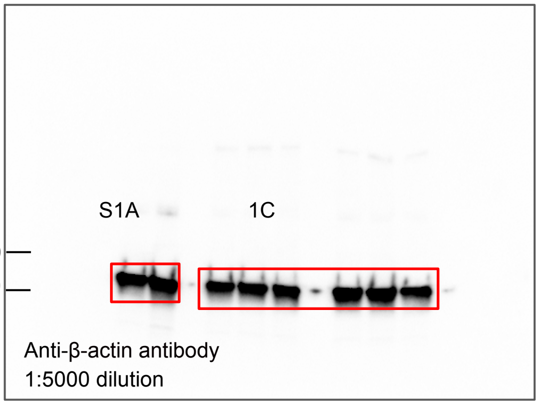

3. Full unedited gel for Figure 3B

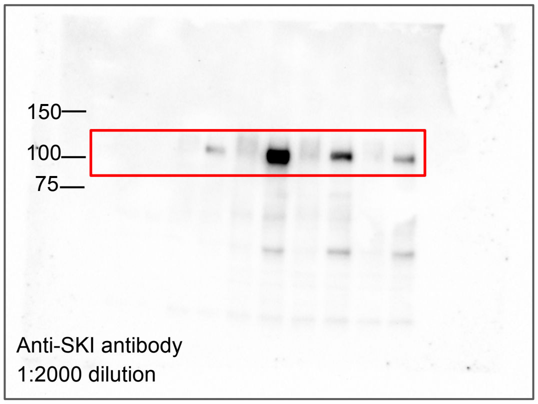

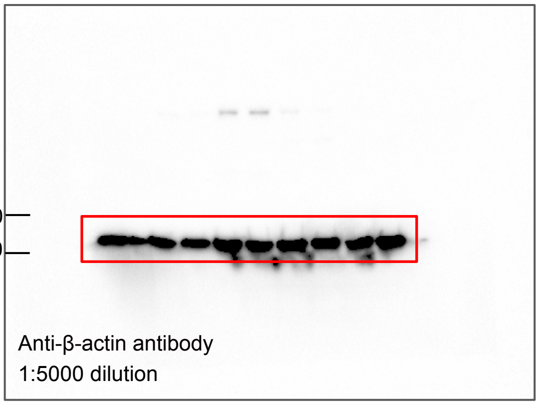

4. Full unedited gel for Figure 4C

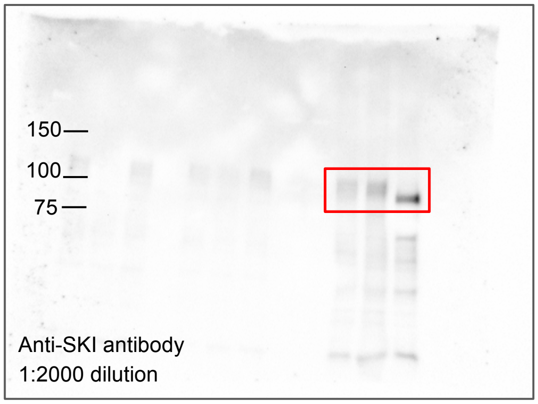

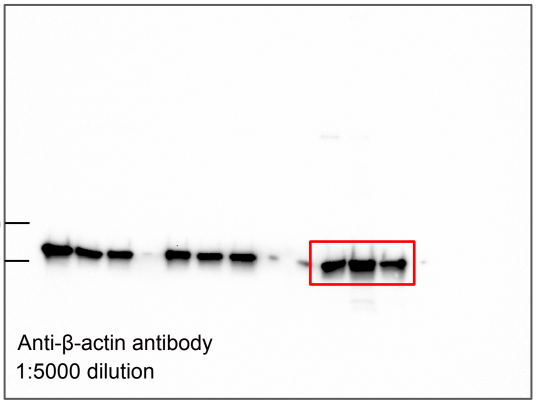

Supplement: Supplementary Figure 2 — Uncropped gel images with size marker indications.This file contains Supplementary Figures 2 : Uncropped western blot images. [file Image_2.pdf]
